# Recent Development of Lysosome-Targeted Organic Fluorescent Probes for Reactive Oxygen Species

**DOI:** 10.3390/molecules28186650

**Published:** 2023-09-15

**Authors:** Van-Nghia Nguyen, Haidong Li

**Affiliations:** 1 Institute of Research and Development, Duy Tan University, Da Nang 550000, Vietnam; 2 School of Computer Science, Duy Tan University, Da Nang 550000, Vietnam; 3 School of Bioengineering, Dalian University of Technology, Dalian 116024, China; lihd@dlut.edu.cn

**Keywords:** fluorescent probes, fluorescence imaging, lysosome, ROS, ROS detection

## Abstract

Reactive oxygen species (ROS) are extremely important for various biological functions. Lysosome plays key roles in cellular metabolism and has been known as the stomach of cells. The abnormalities and malfunctioning of lysosomal function are associated with many diseases. Accordingly, the quantitative monitoring and real-time imaging of ROS in lysosomes are of great interest. In recent years, with the advancement of fluorescence imaging, fluorescent ROS probes have received considerable interest in the biomedical field. Thus far, considerable efforts have been undertaken to create synthetic fluorescent probes for sensing ROS in lysosomes; however, specific review articles on this topic are still lacking. This review provides a general introduction to fluorescence imaging technology, the sensing mechanisms of fluorescent probes, lysosomes, and design strategies for lysosome-targetable fluorescent ROS probes. In addition, the latest advancements in organic small-molecule fluorescent probes for ROS detection within lysosomes are discussed. Finally, the main challenges and future perspectives for developing effective lysosome-targetable fluorescent ROS probes for biomedical applications are presented.

## 1. Introduction

### 1.1. Fluorescence Imaging and Fluorescent Probes

Intravital imaging technology is a crucial approach for modern biology and the medical sciences, and allows us to gain a better insight into the physiological activities of living organisms at the molecular level [1,2,3]. Among various imaging techniques, fluorescence imaging shows significant potential in biomedical science due to its unique advantages such as noninvasiveness, high spatial resolution and sensitivity, fast response time, technical simplicity, and the lack of ionizing radiation exposure [2,4,5,6,7]. In recent decades, significant advancements in fluorescence imaging technology have expanded the possibilities for biological imaging. Ratiometric fluorescence imaging offers distinct advantages in contrast to fluorescence-intensity-based imaging, primarily due to its built-in self-calibration and robust anti-interference capabilities [8]. In addition, high-speed, high-resolution, and field-of-view (FOV) fluorescence imaging offers additional advantages, enabling biologists to utilize a diverse range of models for investigating biological systems [9,10].

**Figure 1:**
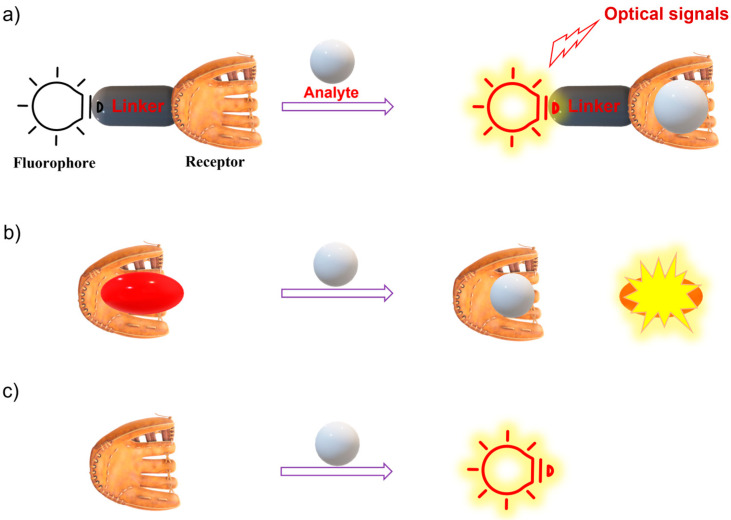
Three main sensing mechanisms of fluorescent chemical sensors. (**a**) Binding site-signaling subunit approach (reversible recognition); (**b**) Displacement approach; (**c**) Chemodosimeter (reaction-based fluorescent probe).

Since the key component of the fluorescence imaging technique is the fluorescent probe, advanced smart fluorescent probes are urgently required for it to fully be applied in clinical practice. A fluorescent probe is defined as a material that can interact with a target and transfer the recognition event into an optical signal output. A fluorescent probe typically involves a recognition site, a fluorophore, and a sensing mechanism [11,12,13]. Typically, there are three types of fluorescent probes, including type 1: the binding-site-signaling subunit approach on the basis of molecular recognition and host–guest chemistry; type 2: a displacement approach relying on a coordination complex; and type 3: chemodosimeters (reaction-based fluorescent probes) (Figure 1) [4,14,15]. Thus far, many fluorescent chemical sensors have been developed for biosensing and bioimaging. In this regard, various photophysical strategies have been established to design fluorescent probes, including photo-induced electron transfer (PET) [16,17,18], chelation-induced fluorescence enhancement (CHFE), Forster resonance energy transfer (FRET) [11,19], intramolecular charge transfer (ICT) [20,21,22], excited-state intramolecular proton transfer (ESIPT) [23,24,25,26], monomer–excimer systems [11,19], and aggregation-induced enhancement (AIE) [27,28,29].

**Figure 2:**
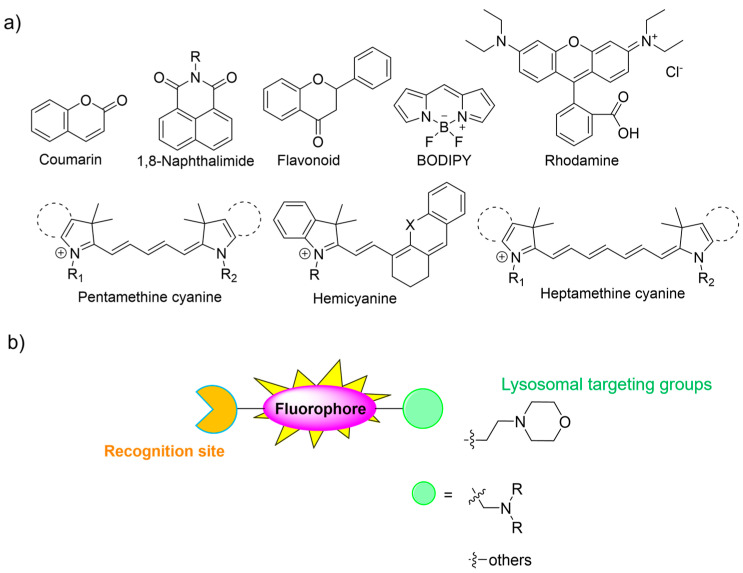
(**a**) Conventional fluorophore platforms and (**b**) general structures of lysosome-targeted fluorescent chemical probes. The different background colors in the Figure indicate the different parts of the structure of the fluorescent probe.

Up to now, most fluorescent ROS probes have belonged to type 3, chemodosimeters, which are designed and developed on the basis of various conventional fluorophore skeletons such as coumarin, naphthalimide, flavonoid, BODIPY, rhodamine, pentamethine cyanine, hemicyanine, and heptamethine cyanine (Figure 2a) [4,11,30,31,32,33]. Generally, the consideration of a “turn-on” or ratiometric fluorescent probe is more desirable compared to “turn-off” fluorescent types due to its excellent sensitivity [34,35,36]. In addition, near-infrared (NIR) fluorescent probes are of great interest because they can improve the penetration depth of imaging and the signal-to-noise ratio in fluorescence imaging [2]. This review highlights the design approaches of small-molecule fluorescent probes and their bio-applications for biosensing and bioimaging of ROS in lysosomes. We believe that this review may encourage more and more researchers to develop smart chemical probes for practical applications.

### 1.2. Lysosomes and Lysosome-Targeted Fluorescent Probes

Lysosomes are small membrane-enclosed cytoplasmic organelles with sizes of 0.1–0.5 µm in diameter, and lysosomes are described as the stomach of cells. Lysosomes are responsible for degrading intracellular biomolecules such as proteins, nucleic acids, lipids, and carbohydrates, triggered by various lysosomal enzymes. Lysosomes have acidic environments with a pH range of 4.0 to 6.0 [37,38]. Although lysosomes play an important role in physiological activities, lysosome dysfunction might induce numerous diseases. Therefore, an in-depth understanding of the lysosomal working principle becomes highly imperative for modern biomedical research [37]. At the cell organelle level, lysosomal ROS play a crucial role in upholding the redox balance of lysosomal functions. However, abnormal concentrations of ROS in lysosomes may induce a loss of lysosomal function. Therefore, it is highly demanded to develop effective fluorescent probes for lysosomal ROS [34,39,40,41]. Lysosomes display unique physicochemical and structural characteristics. In particular, their acidic vesicular structures facilitate the accumulation of weakly basic molecules. Some lysosomal trackers with weakly basic properties have been developed and commercialized. Normally, morpholine and other amine groups are extensively utilized as specific lysosome-targeting units [41]. Notably, the acidic tumor microenvironment may enable the selective delivery of lysosome-targetable probes to tumors based on pH, with the assistance of morpholine or other amine groups possessing lone pairs of electrons. This review provides the design approaches of small-molecule fluorescent probes and their applications for monitoring ROS in lysosomes.

## 2. Lysosome-Targeted Fluorescent Probes for ROS

ROS are chemically active compounds that are naturally produced within living organisms as by-products of oxygen metabolism. ROS encompass various types and play crucial roles in many physiological processes [4,42,43]. However, abnormal ROS generation is closely linked to the pathological processes of various diseases [41]. Consequently, there is a high demand for developing smart fluorescent probes for ROS. This section will introduce the application of small-molecule fluorescent probes for the specific detection of ROS, particularly in lysosomes.

### 2.1. Hydrogen Peroxide (H_2_O_2_)

**Figure 3:**
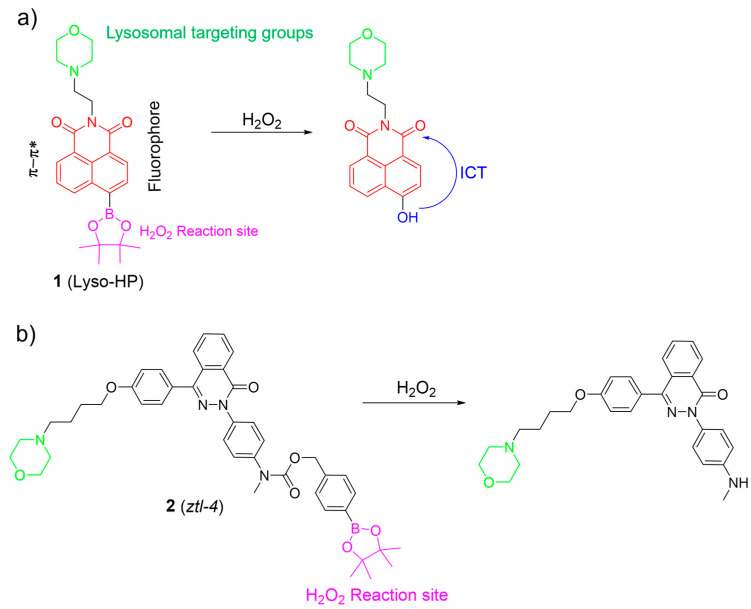
The fluorescence mechanism of **1** (**a**) and **2** (**b**) in response to H_2_O_2_. The different background colors in the Figure indicate the different parts of the structure of the fluorescent probe.

H_2_O_2_ is one of the most significant ROS, and plays a crucial role in redox signaling and oxidative stress in lysosomes. Nevertheless, uncontrolled levels of H_2_O_2_ may cause many diseases, such as neurodegenerative diseases and cancer [44,45,46,47]. Thus, the sensing and imaging of physiological H_2_O_2_ in lysosomes have attracted considerable attention. In this section, organic fluorescent probes based on small molecules for lysosomal H_2_O_2_ are discussed. Lysosome-targeted fluorescent H_2_O_2_ probes usually consist of a boronate ester as the specific sensing unit, a morpholine/amine group as the lysosomal targeting moiety, and a fluorophore. Recently, Lin et al. introduced a two-photon probe, **1**, to detect H_2_O_2_ in lysosomes (Figure 3a) [6]. Probe **1** featured an “acceptor-п-acceptor” electronic structure, initially exhibiting weak fluorescence. Upon exposure to H_2_O_2_, the boronate ester group in **1** underwent oxidation, transforming into an electron-rich hydroxyl group. The transformation triggered a significant increase in fluorescence around 550 nm due to ICT from the hydroxyl group donor to the naphthalimide acceptor. Probe **1** remained unaltered in the presence of other analytes, showcasing its exceptional selectivity for H_2_O_2_. Probe **1** was successfully employed to visualize lysosomal H_2_O_2_ in cells and tissues using two-photon fluorescence imaging.

Based on the phthalazinone scaffold, Xu et al. developed a lysosome-targetable two-photon probe, **2**, for H_2_O_2_. Probe **2** was specifically designed to investigate the dynamic progression of hypoxia–reoxygenation injury (Figure 3b) [48]. Probe **2** demonstrated rapid responsiveness, high sensitivity, and excellent selectivity towards H_2_O_2_. By incorporating the morpholine group, **2** was effectively utilized to monitor H_2_O_2_ within lysosomes. The investigation revealed that reoxygenation potentially led to the accumulation of H_2_O_2_ in lysosomes among post-hypoxia cells.

**Figure 4:**
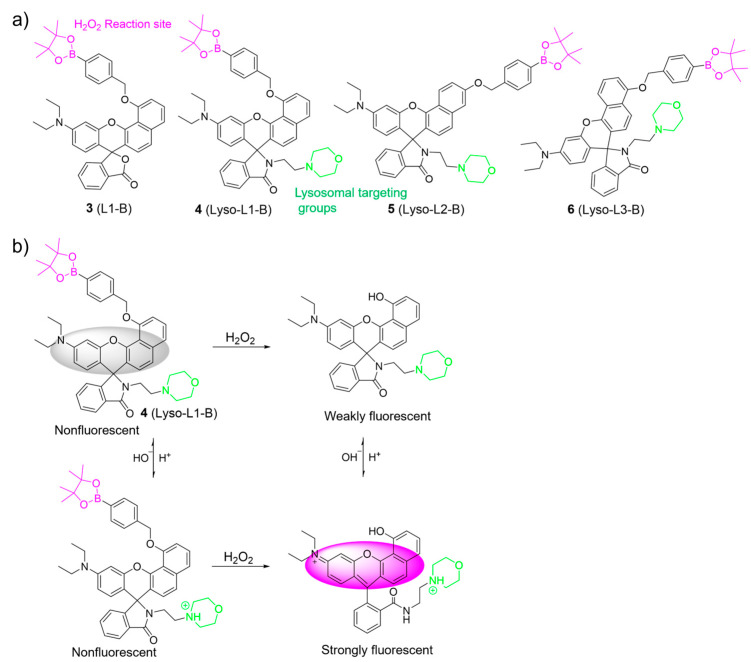
(**a**) Chemical structures of **3**–**6**. (**b**) The sensing mechanism for H_2_O_2_ of **4**. The different background colors in the Figure indicate the different parts of the structure of the fluorescent probe.

Employing a similar oxidative cleavage of the boronate ester strategy, Zhao et al. developed pH-activatable probes **3**–**6** for sensing H_2_O_2_ in lysosomes (Figure 4) [49]. In their work, an aryl boronate group, a morpholine moiety, and a benzorhodol fluorophore were used as the H_2_O_2_-sensing moiety, the lysosome-locating moiety, and the pH-responsive unit, respectively. The aryl boronate moiety was introduced into the different positions of the benzorhodol fluorophore to evaluate the response of the probes to H_2_O_2_. Among them, **4** exhibited an excellent ability to selectively monitor H_2_O_2_ under lysosomal pH (4.5–5.0) compared to **5** and **6**. Cell imaging studies further demonstrated that **4** was able to image endogenous H_2_O_2_ in the lysosomes of living cells.

**Figure 5:**
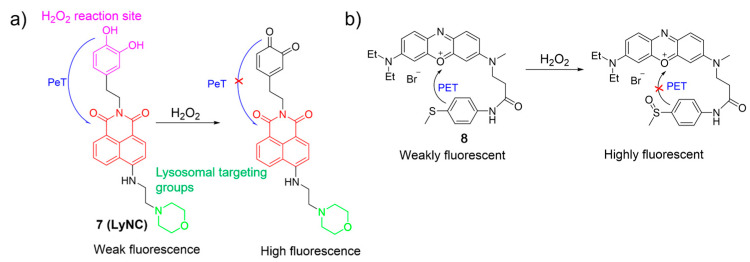
The proposed mechanism for the fluorescence response of **7** (**a**) and **8** (**b**) to H_2_O_2_. The different background colors in the Figure indicate the different parts of the structure of the fluorescent probe. “X” represent for “prohibition”.

Kumar et al. constructed a fluorescent probe, **7**, for monitoring H_2_O_2_, using a naphthalimide as the fluorophore, a catechol as the reactive site, and a morpholine moiety as the lysosome-targeting group (Figure 5a) [50]. In the presence of H_2_O_2_, the probe converted its catechol moiety into an *o*-quinone form, activating emission by blocking PeT from the catechol to the naphthalimide core. Probe **7** was sensitive and selective towards H_2_O_2_, and served as a promising fluorescence imaging agent for tracking H_2_O_2_ levels in lysosomes, brain tissues, and living nematodes.

Ge et al. reported an NIR lysosome-targetable probe, **8**, using phenoxazinium and methyl(phenyl)sulfane as the fluorophore and the sensing unit, respectively, for H_2_O_2_ detection (Figure 5b) [51]. With the addition of H_2_O_2_, oxidation took place at a sulfur atom to produce the corresponding sulfoxide, which led to an increase in fluorescence intensity around 676 nm by the suppression of the PET process. Probe **8** showed good selectivity, excellent sensitivity, and rapid response time. Cell imaging studies indicated that **8** had a potential for detecting and visualizing lysosomal H_2_O_2_ in cells.

**Figure 6:**
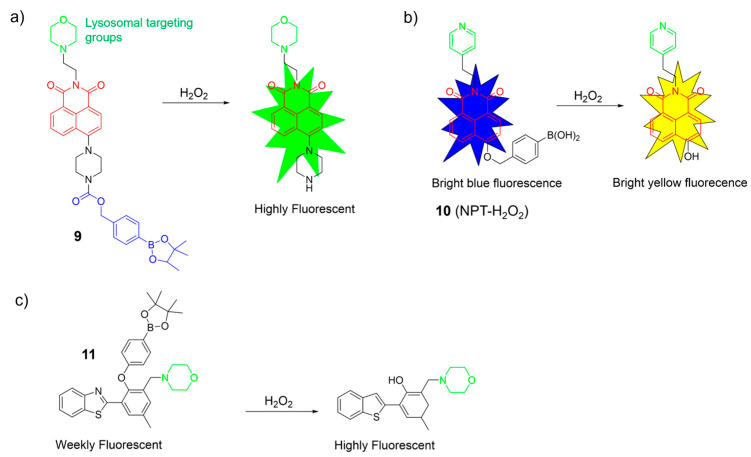
Sensing mechanism of fluorescent probes **9**–**11** (**a**–**c**) for monitoring H_2_O_2_. The different background colors in the Figure indicate the different parts of the structure of the fluorescent probe.

Yoon et al. synthesized a boronate-based H_2_O_2_ probe, **9**, using a naphthalimide as the fluorophore and a morpholine moiety as the targeting group (Figure 6a) [52]. Probe **9** exhibited remarkable specificity for H_2_O_2_, both in the solution and the living cells. The cellular imaging indicated its potential for monitoring endogenous and exogenous H_2_O_2_ levels effectively. Furthermore, time-dependent fluorescence bioimaging provided additional verification of the probe’s efficacy as a reliable marker for detecting H_2_O_2_.

Peng et al. introduced a ratiometric fluorescent probe, **10**, for H_2_O_2_, utilizing a naphthalimide as the fluorophore, a benzyl boronic acid as the reactive moiety for H_2_O_2_, and a pyridine group as the lysosomal targeting unit (Figure 6b) [53]. Without H_2_O_2_, it emitted bright blue light. With H_2_O_2_, it emitted vivid yellow light. Notably, probe **11** exhibited excellent sensing performance for H_2_O_2_: (1) it displayed a target-responsive ratiometric fluorescence; (2) it featured a significant Stokes shift with distinct 425 nm and 550 nm channels; and (3) it responded rapidly (<1 min) and selectively to H_2_O_2_ over other substances. Probe **11** successfully detected and imaged H_2_O_2_ in biological systems.

Ma et al. developed a H_2_O_2_-specific probe, **11**, using a benzothiazole framework as the fluorophore, a boric acid ester as the reactive site, and a morpholine group as the lysosome-targeted unit (Figure 6c) [54]. In the absence of H_2_O_2_, the probe showed weak fluorescence. However, the fluorescence intensity significantly increased in the presence of H_2_O_2_. Probe **11** displayed a good linear correlation with a low LOD of 0.46 μM, indicating excellent sensitivity. Probe **11** was non-toxic and effectively imaged H_2_O_2_ in the lysosomes of A549 cells.

### 2.2. Hypochlorous Acid (HOCl)

**Figure 7:**
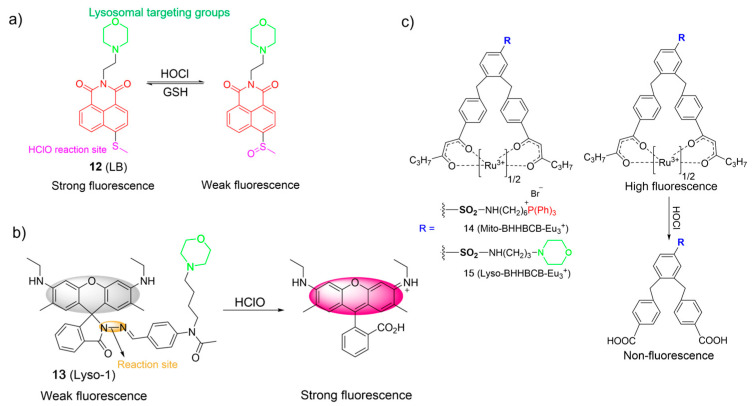
The chemical structures and proposed mechanism for the fluorescence response of **12**–**15** (**a**–**c**) towards HOCl. The different background colors in the Figure indicate the different parts of the structure of the fluorescent probe.

Hypochlorous acid (HOCl) is also known as a crucial ROS in living systems, and is mainly generated through the reaction of chloride (Cl¯) and H_2_O_2_ in the presence of myeloperoxidase (MPO) [4,34,55,56]. HOCl plays a pivotal role within the context of the immune system. However, excessive HOCl production is implicated in numerous pathological processes, leading to various diseases, even cancer [39,41,56]. Accordingly, the consideration of probes for lysosomal HOCl has garnered great interest. In 2017, Ye et al. introduced a two-photon fluorescent probe, **12**, based on the ICT mechanism for detecting HOCl. Probe **12** was prepared via a direct attachment of the methyl thioether group to the naphthalimide structure at its four-position (Figure 7a) [57]. Upon the addition of ClO¯ into solutions of **12**, the absorption peak at 405 nm diminished as the color of the solutions changed from yellow to colorless, and the emission band around 505 nm was gradually quenched. The observation was ascribed to the conversion of methyl thioether into sulfoxide through oxidative processes. Probe **12** exhibited a low LOD of 0.674 µM and a rapid response time in a broad working pH range (pH 4.0 to pH 10.0). In addition, **12** was able to monitor the redox cycles between ClO¯ and GSH because the resulting oxidized product could be reconverted to **12** by GSH. Finally, probe **12** was used to image HOCl in lysosomes by using one- and two-photon fluorescence imaging.

Zeng et al. developed a water-soluble probe, **13**, for determining HOCl using a hydrazone as the responsive unit and a morpholine as the lysosome-targeting moiety (Figure 7b) [58]. In the absence of HOCl in water, **13** was weakly fluorescent (*Φ*_PL_ = 0.04). The emission of **13** rapidly grew in intensity (*Φ*_PL_ = 0.77) when ClO¯ was added. The titration outcomes revealed a highly linear association between fluorescence intensities and ClO¯ concentrations (0.5−2.5 μM) and the low LOD (~60 nM). The “off-on” sensing mechanism was achieved through the promotion of hydrazone oxidation by ClO¯ and led to the ring-opening of the spirolactame. Probe **13** was successfully employed for imaging of HOCl in lysosomes.

Yuan et al. designed two europium-complex-based probes for HOCl, in which a triphenylphosphonium (**14**) and a morpholine (**15**) were used as the mitochondrial targeting moiety and the lysosomal targeting moiety, respectively (Figure 7c) [59]. Upon the addition of incremental amounts of HOCl, the carbonyl group of Eu^3+^ complexes readily transformed into a carboxylic acid. Consequently, the luminescence intensity of probes gradually quenched due to the decomposition of complexes. They displayed remarkable sensitivity (<15 nM) in a wide pH range and a rapid response (<5 s). Both complexes were applied to visualize HOCl in mitochondrial and lysosomal cells and animals using time-gated luminescence microscopy.

**Figure 8:**
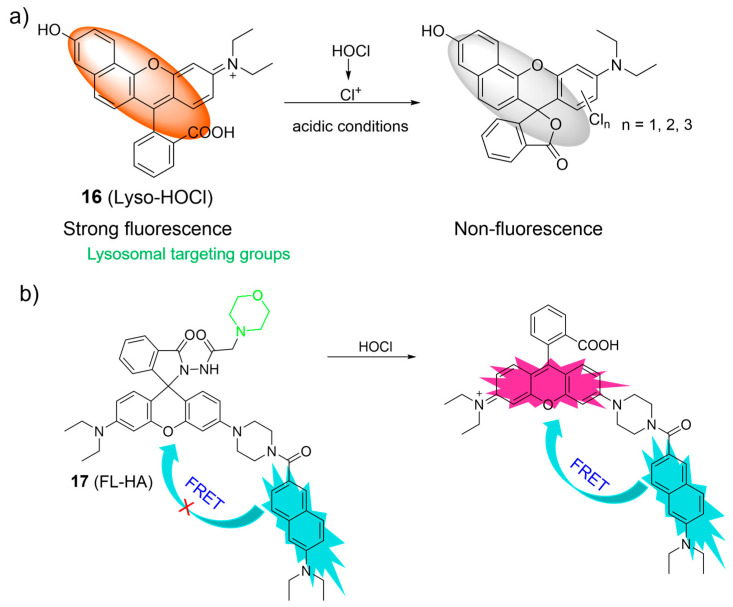
The proposed sensing mechanism for the fluorescence response of **16** (**a**) and **17** (**b**) to HOCl. The different background colors in the Figure indicate the different parts of the structure of the fluorescent probe. “X” represent for “prohibition”.

Considering the distinct chlorination-induced cyclization properties of rhodamine acid, Zhang et al. reported a pH-mediated probe, **16**, for the recognition of HOCl (Figure 8a) [60]. Upon the introduction of HOCl into the acidic solution of **16**, HOCl was easily decomposed into chlorinium ions (Cl^+^). The resulting Cl^+^ induced the ring-closure process of the rhodamine derivative to form a chlorinated spirolactone structure. As a result, a significant decrease in fluorescence at 587 nm was observed. Probe **16** was reported to be highly sensitive (at the picomolar level) and selective over other bioactive molecules. Probe **16** was applied for imaging of HOCl in the lysosomes of live RAW264.7 macrophage cells.

A ratiometric fluorescent probe, **17**, for visualizing HOCl, relying on the FRET mechanism and the rhodamine ring-opening processes, was developed by Lin et al. (Figure 8b) [61]. The probe was composed of a naphthalene as the donor, a rhodamine fluorophore as the acceptor, and a morpholine as the lysosome-targeting unit. As shown in Figure 8b, the rhodamine ring-opening processes were induced upon the addition of HOCl to the **17** solutions, resulting in the ratiometric fluorescence response. Probe **17** was used for ratiometric imaging of lysosomal HOCl.

**Figure 9:**
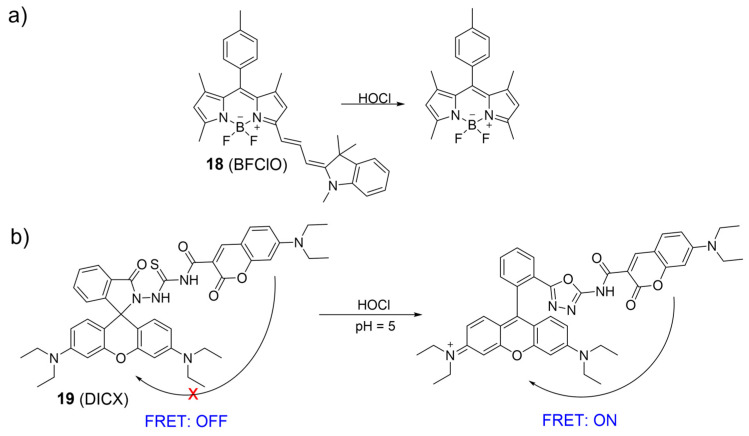
The fluorescence mechanism of **18** (**a**) and **19** (**b**) in response to HOCl. “X” represent for “prohibition”.

An NIR ratiometric fluorescent probe for HOCl was developed by Fan et al., utilizing a BODIPY dye conjugated with Fisher aldehyde (Figure 9a) [62]. During the addition of NaClO into the **18** solutions, the absorption band centered at 650 nm gradually decreased while a new absorption band at 501 nm emerged, resulting in a color shift from blue to vibrant pink. Meanwhile, fluorescent intensity at 713 nm (λ_ex_ = 635 nm) decreased and fluorescent intensity at 511 nm (λ_ex_ = 488 nm) increased with the gradual addition of NaClO. The fluorescence intensity ratio (I_511_ nm/I_713_ nm) correlated well with the NaOCl concentrations and had a 300-fold enhancement in the presence of HOCl. Probe **18** exhibited high sensitivity to HOCl and was utilized to visualize HOCl in lysosomes with low cytotoxicity.

Taking advantage of the ratiometric fluorescent probe, Zhao et al. synthesized a coumarin–rhodamine platform, **19**, for HOCl detection based on the FRET mechanism and rhodamine ring-opening characteristics. Coumarin and rhodamine moieties were linked by monothio-bishydrazide (Figure 9b) [63]. Without HOCl, the rhodamine moiety existed in a ring-closed form, and **19** displayed only the fluorescence of the coumarin moiety at 480 nm under 410 nm excitation. However, upon the addition of HOCl, a decrease in the emission intensity of coumarin and an increase in rhodamine emission were observed. The observation was described as a proficient FRET mechanism, initiated by the HOCl-induced ring-opening process of the rhodamine platform, facilitating energy transfer from the coumarin donor to the rhodamine acceptor. Probe **19** was suitable for sensing lysosomal HOCl.

**Figure 10:**
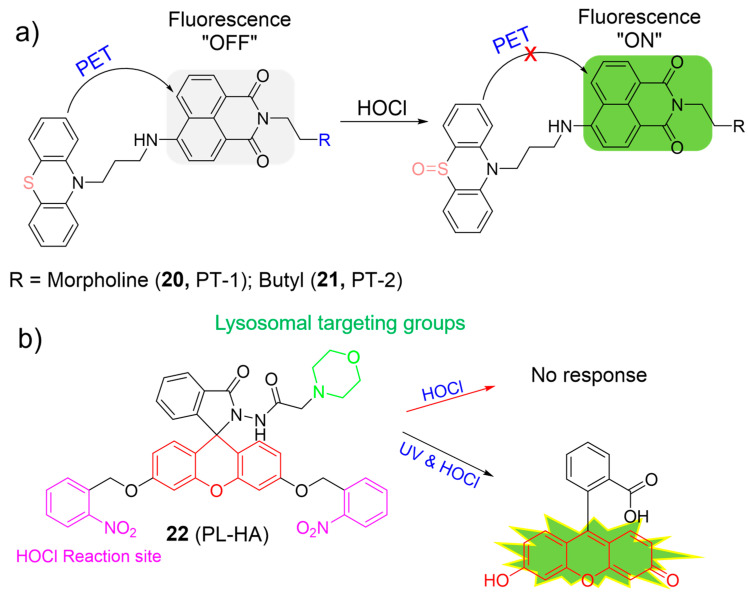
The fluorescence mechanism of **20**–**22** (**a**,**b**) in response to HOCl. The different background colors in the Figure indicate the different parts of the structure of the fluorescent probe. “X” represent for “prohibition”.

Zeng et al. prepared naphthalimide-based “off–on” fluorescent probes (**20** and **21**) for sensing HOCl (Figure 10a) [64]. The intact probes were reported to be essentially non-fluorescent because of the PET process from the phenothiazine (electron donor) to the naphthalimide fluorophore (electron acceptor). Upon the addition of HOCl, the phenothiazine unit was oxidized to form sulfoxide, which inhibited the PET process. As a result, the fluorescence intensity significantly increased over 160-fold and 34-fold for **20** and **21**, respectively. Both probes showed high selectivity and selectivity with fast response time (within 10 s) towards HOCl. They were applied to monitor HOCl in lysosomes. In particular, **21** was capable of accurately exploring the functions of lysosomal HOCl in cells.

Considering both photocaging technology and traditional lysosomal HOCl-responsive systems, Lin et al. developed the first photo-controllable fluorescent probe for HOCl, using a 2-nitrobenzyl group as the photon-sensitive moiety, a morpholine as the targeting unit, and a fluorescein as the fluorophore (Figure 10b) [65]. Probe **22** itself exhibited weak fluorescence (*Φ*_FL_ = 0.03) and was intact towards HOCl in dark conditions. However, in the presence of HOCl and UV light irradiation, an approximate 102-fold enhancement of fluorescence intensity (*Φ*_FL_ = 0.57) was observed under neutral and acidic conditions, indicating the formation of fluorescein dye. Probe **22** was highly sensitive (62 nM) and selective for HOCl. Probe **22** was suitable for the visualization of HOCl in lysosomes after UV-light photolysis.

**Figure 11:**
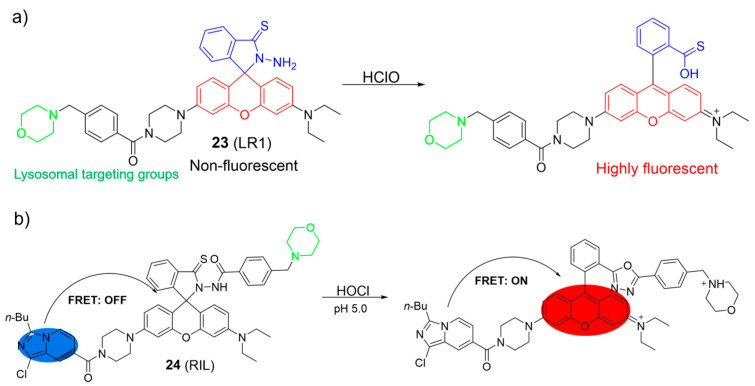
Chemical structures and the proposed detection mechanism of **23** (**a**) and **24** (**b**) toward HOCl. The different background colors in the Figure indicate the different parts of the structure of the fluorescent probe.

Cao et al. reported a rhodamine-based HOCl fluorescent probe, **23** (Figure 11a) [66]. In the presence of HOCl, **23** rapidly reacted with HOCl, leading to an enhancement of fluorescence at 582 nm. In addition, a clear change in the solution color from colorless to pink was observed with the naked eye. The phenomena were attributed to the structural conversion of the rhodamine moiety from the spirocyclic form to the ring-opened form by HOCl. The LOD of **23** was 2.6 nM. Probe **23** could detect endogenous HOCl in lysosomes with low cytotoxicity.

Cao et al. developed a FRET-based system as a ratiometric fluorescent sensor for HOCl using an imidazo[1,5-a]pyridine and a rhodamine (Figure 11b) [67]. When HOCl was introduced, the absorption peak at 360 nm for imidazo[1,5-a]pyridine remained nearly unchanged but a new absorption peak at 565 nm emerged, resulting in a shift in color from colorless to light red. The emission peak of the imidazo[1,5-a]pyridine decreased significantly, while a rhodamine-specific emission peak at 588 nm grew with HOCl addition. Probe **24** exhibited a rapid response to HOCl, excellent selectivity, and a low LOD of 27 nM. The fluorescence imaging demonstrated that **24** enabled visualizations of endogenous HOCl in lysosomes.

**Figure 12:**
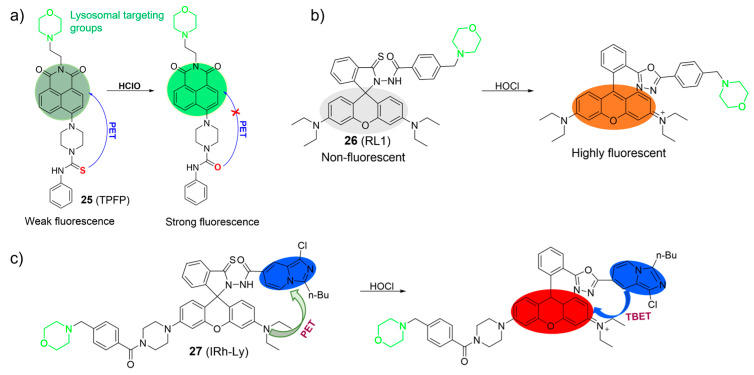
The proposed sensing mechanism of **25**–**27** (**a**–**c**) toward HOCl. The different background colors in the Figure indicate the different parts of the structure of the fluorescent probe. “X” represent for “prohibition”.

Chen et al. developed a PET-based two-photon fluorescent probe, **25**, for lysosomal HOCl detection using a naphthalimide as the fluorophore, a phenyl-thiourea as the HOCl recognition unit, and a morpholine as the lysosome-targetable group (Figure 12a) [68]. Probe **25** exhibited weak emission (*Φ*_PL_ < 0.01) in the absence of HOCl. However, upon the addition of HOCl, the fluorescence significantly increased. The enhanced fluorescence intensity was primarily attributed to the oxidation of thiourea to urea triggered by HOCl. The LOD of **25** was estimated to be 5.7 nM. Probe **25** was capable of imaging HOCl using one- and two-photon microscopy.

Similarly, Cao et al. also described a fluorescent probe, **26**, based on the rhodamine fluorophore for HOCl (Figure 12b) [69]. When probe **26** reacted with HOCl, it resulted in the emergence of an absorption band at 568 nm, accompanied by a noticeable change in the solution’s color from colorless to pink. This phenomenon allowed for visual, colorimetric detection of HOCl with the naked eye. Furthermore, an increase in fluorescence intensity at 592 nm was observed following the addition of HOCl. The observation was attributed to the structural transformation from the spirocyclic form to the ring-opened configuration of the rhodamine derivative. Probe **26** demonstrated excellent selectivity and sensitivity to HOCl (LOD = 2.8 nM) in aqueous conditions. Probe **26** was successfully applied to image endogenous HOCl in RAW264.7 cells.

In their ongoing research in this field, Shen et al. utilized the rhodamine-imidazo[1,5-a] pyridine platform to develop a through-bond energy transfer (TBET)-based ratiometric fluorescent probe, **27**, for the selective detection of lysosomal HOCl (Figure 12c) [70]. In the absence of HOCl, the rhodamine moiety remained in its non-fluorescent ring-closed form, and probe **27** exhibited only the typical absorption and fluorescence of the imidazo[1,5-a] pyridine moiety. However, upon interaction with HOCl, an absorption band in the range of 500−600 nm and a fluorescence peak corresponding to the ring-opened rhodamine emerged. The spectral change was attributed to the formation of the oxadiazole compound, facilitated by efficient TBET between the imidazo[1,5-a] pyridine donor and the rhodamine acceptor. Probe **27** was utilized to image HOCl in lysosomes.

**Figure 13:**
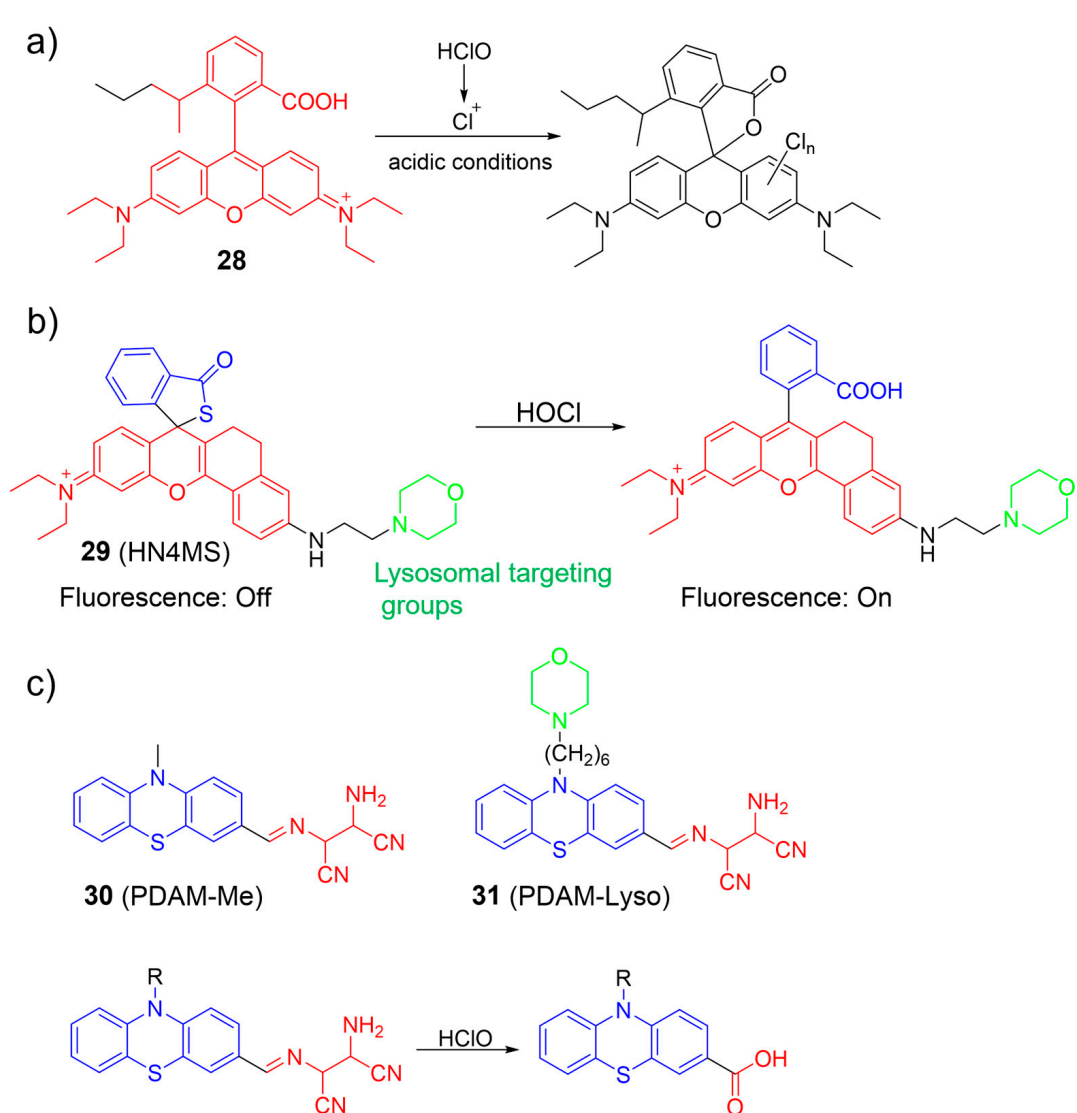
The fluorescence response of **28**–**31** (**a**–**c**) to HOCl. The different background colors in the Figure indicate the different parts of the structure of the fluorescent prob.

Zhang et al. proposed an AIE-based ratiometric fluorescent nanoprobe, **28**, primarily composed of an AIE fluorogen and a rhodamine B unit for imaging HOCl in living cells (Figure 13a) [71]. The nanoprobe exhibited excellent water solubility, high photostability, and good biocompatibility. In the presence of ClO^−^ under acidic conditions, the rhodamine B moiety was converted into a chlorinated spirolactone structure. The transformation led to a significant decrease in the fluorescence intensity of the rhodamine B unit and minimal change in the fluorescence intensity of the AIE dye, resulting in a shift in emission color from orange to blue. Moreover, nanoprobe **28** was applied to monitor HOCl within lysosomes.

A rhodamine-based spiro-ring platform, **29**, was utilized by Gong et al. for HOCl detection by connecting a thiolactone rhodamine unit with a morpholine lysosome-specific moiety (Figure 13b) [72]. When exposed to HOCl, notable photophysical changes were observed, including a gradual increase in fluorescence emission (λ_em_ = 632 nm) and absorption (λ_ex_ = 591 nm). The observation was attributed to the rapid chlorination reaction between HOCl and the sulfur atom, resulting in the spiro-ring-opening process. Probe **29** was utilized to image lysosomal HOCl in cells and tissues using two-photon microscopy.

Wang et al. developed phenothiazine-based ratiometric fluorescent probes (**30** and **31**) for sensing HOCl by converting AIE into ICT emission in solution (Figure 13c) [73]. In an aqueous medium, intramolecular motion was suppressed, leading to highly red ICT emission by the probes. Upon the addition of HOCl, the emission peak at 620 nm was reduced, while the emission intensity at 470 nm increased. Consequently, the fluorescence intensity ratios (I470/I620) increased about 600-fold. The observed phenomenon was attributed to the selective cleavage of the imine linkage between the phenothiazine and the diaminomaleonitrile (DAMN) in the probes by HOCl, resulting in the removal of the DAMN moiety and the eventual formation of aldehyde or carboxylic acid products. Imaging results demonstrated that **31** was more effective in monitoring endogenous HOCl than a non-targeting probe. Probe **31** was successfully utilized to monitor HOCl generation in zebrafish.

**Figure 14:**
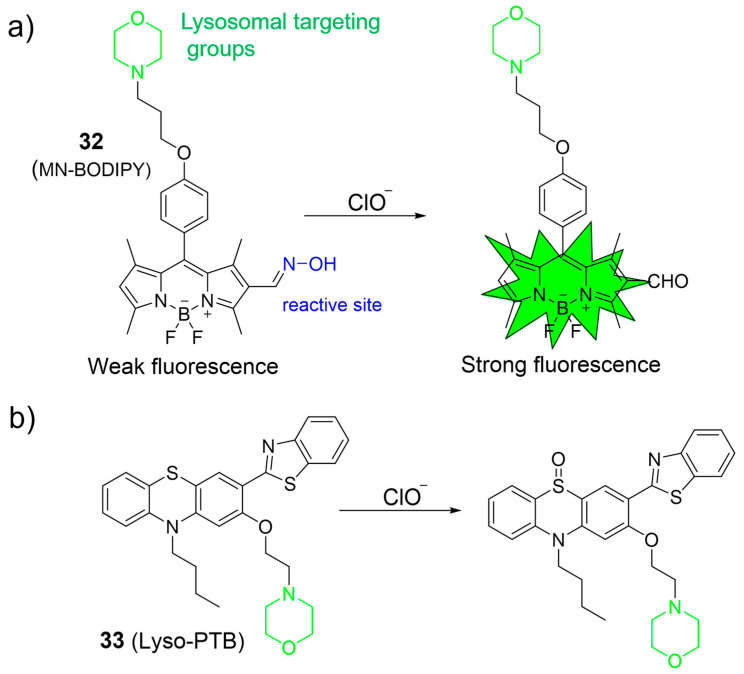
The detection mechanism of **32** (**a**) and **33** (**b**) towards HOCl. The different background colors in the Figure indicate the different parts of the structure of the fluorescent probe.

Qian et al. introduced a lysosome-targeting fluorescent probe, **32**, by incorporating an aldoxime unit into the BODIPY fluorophore platform for specific ClO¯ detection (Figure 14a) [74]. Upon exposure to ClO¯, the probe exhibited a substantial 29-fold increase in fluorescence intensity at 530 nm, which was ascribed to the conversion of aldoximes into aldehydes through oxidation. Probe **33** displayed an LOD of 16.5 nM and a rapid response to ClO¯ within 60 s. Notably, **32** was effectively employed to image ClO¯ levels in MCF-7 cells.

Hou et al. devised a novel phenothiazine derivative, **33**, serving as a lysosome-targeted fluorescent probe for detecting ClO¯ (Figure 14b) [75]. Probe **33** combined a lysosome-targeting group (the morpholine), a fluorophore moiety (the phenothiazine-benzo[d]thiazole), and a recognition site (the sulfur atom within phenothiazine). In the recognition process, the sulfur atom in phenothiazine could be readily oxidized by ClO¯ to form a sulfoxide, leading to a substantial blue shift in the emission. The effectiveness of **33** in ClO¯ detection was demonstrated both in cells and in zebrafish.

### 2.3. Hypobromous Acid (HOBr)

**Figure 15:**
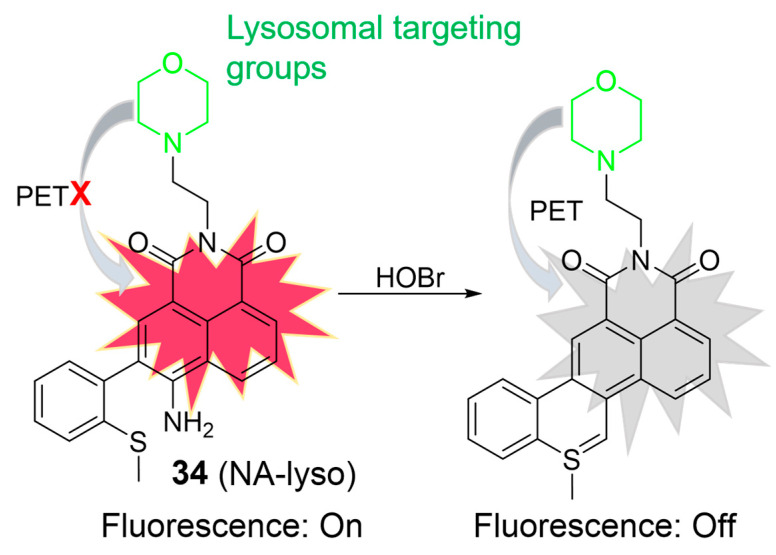
Schematic illustration for the fluorescence response of **34** to HOBr. The different background colors in the Figure indicate the different parts of the structure of the fluorescent probe. “X” represent for “prohibition”.

HOBr is formed through the catalysis of MPO, combining hydrogen peroxide (H_2_O_2_) with bromide ions (Br^¯^). Excessive generation of HOBr can lead to a redox imbalance and the loss of lysosomal function, resulting in tissue damage and diseases [76]. In 2018, Zhang et al. developed a two-photon fluorescent probe, **34**, for HOBr (Figure 15) [77]. When exposed to HOBr, both the amino and methylthio groups underwent a cyclization reaction, forming a nitrogen–sulfur double bond. Consequently, the fluorescence intensity at 540 nm gradually decreased upon the addition of HOBr. The “on-off” fluorescence phenomenon was elucidated based on the PET mechanism. The LOD of **34** was determined to be 33.5 nM, indicating good sensitivity. Probe **34** demonstrated its capability to detect HOBr within lysosomes in HeLa cells and visualize endogenous HOBr in living mice using two-photon fluorescence microscopy.

## 3. Conclusions and Outlook

Numerous organic lysosome-targetable probes based on small molecules have been developed for ROS detection, which are summarized in this work. Despite the progress made to date in developing organic small-molecule fluorescent probes for sensing ROS in lysosomes, several issues should be overcome. Firstly, morpholine and other amine-based lysosome-targeting probes can potentially lead to toxicity due to their alkalinizing effect on lysosomal pH. For instance, the alkalinization of lysosomal pH can disrupt the proper functioning of lysosomes, impairing enzyme activity and hindering cellular waste degradation [78,79]. To mitigate the potential toxicities associated with these probes, researchers should meticulously optimize their experimental conditions, including probe concentrations and exposure times. Additionally, proper validation of lysosomal targeting and probe specificity are essential. Secondly, current probes suffer from poor water solubility, poor aqueous stability, and aggregation-induced quenching effects in aqueous media, which limit their performance in biological systems. By considering water-soluble and non-aggregating fluorophore skeletons or employing nanodelivery systems, these drawbacks can be addressed to enhance in vivo imaging.

In addition, fluorescent probes based on coumarin, naphthalimide, flavonoid, BODIPY, and rhodamine skeletons usually exhibit short excitation (<550 nm)/emission (<600 nm) wavelengths, limiting their applications in in vivo imaging due to restricted penetration depth, the signal-to-noise ratio in fluorescence imaging, and the risk of phototoxicity. To address these issues, the extension of fluorophore-skeleton-based probes for NIR absorption and emission and the consideration of two-photon fluorescent probes from these skeletons are crucial. Currently, most of the NIR fluorescent probes used for in vivo imaging are cyanine derivatives. An exemplary case is indocyanine green (ICG), which has received clinical approval from the FDA for in vivo imaging applications. Thus, the development of organic lysosome-targetable ROS probes based on cyanine platforms should be considered. However, the enhancement of the performance of cyanine-based probes is still required due to the high susceptibility of cyanine skeletons to oxidants and photobleaching. Nanotechnology can be used to address these limitations of cyanine-based probes. The recent discovery of the hemicyanine platform with superior performance may provide an excellent alternative platform for the construction of lysosome-targetable ROS probes.

Most importantly, the focus should be on developing lysosomal fluorescent ROS probes with clinical potential and ensuring their entry into clinical trials for successful translation. We anticipate that, through the combined efforts of chemists, physicists, biologists, and medical scientists, these challenges will be addressed soon, and organic small-molecule fluorescent probes will serve as powerful molecular tools for gaining a better understanding of the physiological functions of ROS in lysosomes.
